# Comparison of Accu Chek Inform II point-of-care test blood glucose meter with Hexokinase Plasma method for a diabetes mellitus population during surgery under general anesthesia

**DOI:** 10.1007/s10877-021-00656-6

**Published:** 2021-01-26

**Authors:** B. Lascaris, H. G. Freling, M. A. Edens, M. J. Fokkert, C. G. Olthof, R. J. Slingerland

**Affiliations:** 1 Department of Anesthesiology, University Medical Center Groningen, Hanzeplein 1, 9713 GZ Groningen, The Netherlands; 2 Department of Innovation and Science, Isala Zwolle, Zwolle, the Netherlands; 3 Department of Clinical Chemistry Laboratory, Isala Zwolle, Zwolle, the Netherlands; 4 Department of Anesthesiology, Isala Zwolle, Zwolle, the Netherlands

**Keywords:** Point-of-care systems, General anesthesiology, Blood glucose, Monitoring

## Abstract

**Purpose:**

Blood glucose (BG) concentrations of patients with diabetes mellitus (DM) are monitored during surgery to prevent hypo- and hyperglycemia. Access to point-of-care test (POCT) glucose meters at an operating room will usually provide monitoring at shorter intervals and may improve glycemic control. However, these meters are not validated for patients under general anesthesia.

**Methods:**

This cross-sectional study included 75 arterial BG measurements from 75 patients (71 with DM, mostly insulin dependent) who underwent elective non-cardiac surgery under general anesthesia. Arterial blood samples were taken at least 60 minutes after induction. One drop of blood was used for Accu Chek Inform II (ACI II) POCT BG meter and the residual blood was sent to the clinical laboratory for a Hexokinase Plasma reference method. A Bland–Altman plot was used to visualize the differences between both methods, and correlation was assessed using the intra-class correlation coefficient (ICC).

**Results:**

The results showed an estimated mean difference of 0.8 mmol/L between ACI II and the reference method, with limits of agreement equal to -0.6 and 2.2 mmol/L. In general, the reference method produced higher values than ACI II. ICC was 0.955 (95% CI 0.634–0.986), P < 0.001, and concordance correlation coefficient (CCC) was 0.955 (95% CI 0.933–0.970).

**Conclusion:**

Arterial BG measurements during surgery in patients with DM under general anesthesia using POCT BG meter are in general lower than laboratory measurements, but the ICC and CCC show a clinically acceptable correlation. We conclude that POCT measurements conducted on arterial specimens using the ACI II provide sufficiently accurate results for glucose measurement during surgery under general anesthesia.

## Introduction

Blood glucose (BG) monitoring in diabetic patients during general anesthesia is important. Hypoglycemia and hyperglycemia are unwanted conditions perioperatively, with BG concentrations between 4 and 12 mmol/L recommended by most guidelines [1]. BG concentrations can be measured by several methods, including point-of-care test (POCT) BG meters and laboratory methods. General anesthesia often induces hypotension, which can cause reduction of perfusion and thereby a reduction of blood refreshing, leading to less accurate capillary blood glucose measurement compared to the actual value in the systemic circulation. Furthermore, fluid shifts during surgery are common due to blood loss, fluid administrations, and vasodilatory effect of anesthetics. This can also affect capillary blood sampling, which may result in a lower glucose concentration [2]. Controlled capillary measurements depend on a lot of different factors which are not constant during general anesthesia. Arterial measurements are less disturbed by skin temperature and peripheral circulation. We therefore focus on arterial measurements in this study.

In general, laboratory methods are more accurate and so far, only one blood glucose POCT meter has been shown to produce equivalent results to a laboratory method [3]. Laboratory methods, however, take more time and results can be delayed. POCT BG meters have the advantage that they are easy to use and show results in a very short time frame. POCT blood glucose meters are already used in some hospitals for glucose measurements in patients during general anesthesia, with the assumption that the results are comparable to laboratory methods. However, the accuracy of these meters has not been evaluated in detail for these patients.

In line with the most recent European consensus group guidelines, the analytical performance of diagnostic assays should be aligned to patient outcomes [4]. For a POCT glucose assay the performance should be optimal at thresholds for defining hypoglycemia (≤ 4.0 mmol/L) and hyperglycemia (≥ 12.0 mmol/L) during surgery. The advantage of this approach is that it addresses the influence of analytical performance on clinical outcomes that are relevant to patients. The primary disadvantage is that it is only useful for examinations where the links between the test, the clinical decision-making, and the clinical outcomes, are straightforward and strong. Furthermore, analytical specifications will often be influenced by the current measurement quality; results may vary with the actual test method used, the population studied, as well as the healthcare settings.

The usage of POCT BG meters has been increasing. Their use has already been validated for wards and critically-ill patients (5). However, the validity of POCT BG meters may be significantly affected during intraoperative use because of factors such as a change in blood circulation, the lack of muscle activity, and a changed metabolism from surgical stress. Although not a critically ill population as with previous studies of POCT glucose meters, patients receiving insulin and other glucose modifying medications also require accurate and timely peri-operative glucose monitoring, with a particular emphasis on hypoglycemia prevention [6].

During anesthesia, arterial blood glucose values keeping between sharp defined borders will be better for the outcome of patients [7]. POCT measurements will result in faster blood glucose regulation and therefore we performed this comparison study using clear boundaries to determine if a POCT BG meter is safe for patients under general anesthesia [6].

## Methods

### Study setup and population

#### Study design

We performed a cross-sectional study comparing the blood glucose measurement of the Accu Chek Inform II (ACI II) (Roche, Mannheim, Germany) POCT blood glucose meter (the “index test”) and the clinical laboratory (the “reference test”) in patients who underwent elective non-cardiac surgery under general anesthesia.

### Setting

This study was performed in Isala hospital, a general teaching hospital in Zwolle the Netherlands. Data were collected from March 2016 to August 2018.

### Participants

Seventy-five adult patients who underwent elective non-cardiac surgical procedures were included in the analysis, of which 67 had insulin-dependent diabetes mellitus, four had non-insulin dependent diabetes mellitus, and four had no diabetes mellitus. They all received an arterial catheter, which was used for hemodynamic monitoring. The patients with insulin-dependent diabetes mellitus were on insulin therapy (continuous intravenous insulin aspart (NovoRAPID, Novo Nordisk, Alphen a/d Rijn, the Netherlands)).

### Data sources/measurements

Arterial blood samples were collected at a minimum of 60 minutes after induction of general anesthesia. One drop of blood was used for measurement with the ACI II and the residual blood was sent to the clinical laboratory. Blood was collected in a NaF/EDTA-citrate tube (Vacuette FC MIX blood collection, Greiner, Austria) [8]. Samples in the laboratory were analyzed with the Plasma Hexokinase glucose assay on a Cobas 8000 autoanalyzer (Roche, Mannheim, Germany). Glucose standards applied were taken from the National Institute of Standards and Technology (NIST, Washington, DC, USA). The results using these standards were, at all concentrations, analyzed within 3% deviation of the target values. Time of sampling and body temperature were registered. Performance of the ACI II was checked with Eurotrol (Ede, the Netherlands) CueSee quality control materials (< 3% deviation from target value during the whole study period).

We used the test results from the clinical laboratory to determine if adjustments in insulin therapy were necessary. Samples from the same tube were run in duplicate with the laboratory method. All values were automatically linked to the electronic medical record.

Data regarding glucose values were collected from the electronic medical record. In addition, the type of surgery, the age, gender, American Society of Anesthesiologists (ASA)-classification, and body mass index (BMI) of the patient were collected.

### Categorization

Blood glucose levels were compared by their continuous variable (mmol/L) as well as by their category. Categorization was based on the “glucose-regulation during surgery” protocol of the anesthesiology department of Isala clinic, Zwolle, the Netherlands, which advises on the amount of insulin-dose adjustments.

### Study size

A sample size of at least 50 samples is considered sufficient for a reliable study [9]; 75 patients were included in this cross-sectional study.

### Ethics committee approval

The study was approved by the Isala Research Ethics Committee, Zwolle, the Netherlands on 2 January 2017 and registered under the number: 16.12226.

### Statistical analysis

Continuous variables were summarized by mean (± SD) and categorical data were summarized by n (%).

Various plots were used to visualize the differences between both methods. The concordance correlation coefficient (CCC) and intra-class correlation coefficient (ICC) with 95% confidence interval were calculated. The ICC was performed using a two-way mixed effects, absolute agreement, single-measurement model. Also, an alignment and traceability matrix was made to indicate whether or not there would be differences in treatment policy (i.e. insulin adjustment) if the POCT was used in clinical practice instead of the laboratory measurements. In addition, the weighted Cohen’s Kappa was calculated.

Statistical analysis was performed using MedCalc version 19.0.5 and Statistical Package for Social Sciences (SPSS version 24.0). A significance level of 5% (α = 0.05; two-sided) was used.

## Results

### Participants

Patient characteristics are presented in Table 1.

**Table 1:** Patient characteristics

Characteristics	*n* = 75
Age (mean ± SD) in years	71.6 ± 10.4
Sex, No. (%)	
Men	52 (69)
Women	23 (31)
BMI (mean ± SD) in kg m^−2^	28.2 ± 4.7
DM status, No. (%)	
IDDM	71 (95)
No DM	4 (5)
ASA-classification, No. (%)	
ASA 2	26 (35)
ASA 3	48 (64)
ASA 4	1 (1)
Type of procedure, No. (%)	
Craniotomy	9 (12)
Vascular surgery	45 (60)
Abdominal surgery	13 (17)
Other	8 (11)

*ASA* American Society of Anesthesiologists*; BMI* body mass index*, DM* diabetes mellitus, *IDDM* insulin dependent diabetes mellitus

### Blood samples

Throughout the study, 75 arterial blood samples were collected, of which 71 DM patients, mostly insulin dependent. The results are shown in Table 2. Mean glucose concentrations measured with the POCT ACI II and laboratory hexokinase method measurements were 9.5 ± 3.4 mmol/L versus 10.3 ± 3.7 mmol/L, respectively.

**Table 2:** Results

	All (*n* = 75)
Temperature (mean ± SD) in °C	36.4 ± 0.5
BG POCT (mean ± SD) in mmol/l	9.5 ± 3.4
BG lab (mean ± SD) in mmol/l	10.3 ± 3.7
BG (mean difference ± SD) in mmol/l	0.8 ± 0.7
POCT < lab, No (%)	69 (92)
POCT > lab, No (%)	4 (5)
POCT = lab, No (%)	2 (3)
Hypoglycemia*	
POCT, No. (%)	1 (1)
lab, No. (%)	1 (1)
Hyperglycemia**	
POCT, No. (%)	16 (21)
lab, No. (%)	23 (31)

*BG* blood glucose, *POCT* point-of-care test, *lab* laboratory

^a^Hypoglycemia defined by ≤ 4.0 mmol/L

^b^Hyperglycemia defined by ≥ 12.0 mmol/L

### Comparison of ACI II and laboratory hexokinase method

#### Blood glucose in mmol/L

**Fig. 1:**
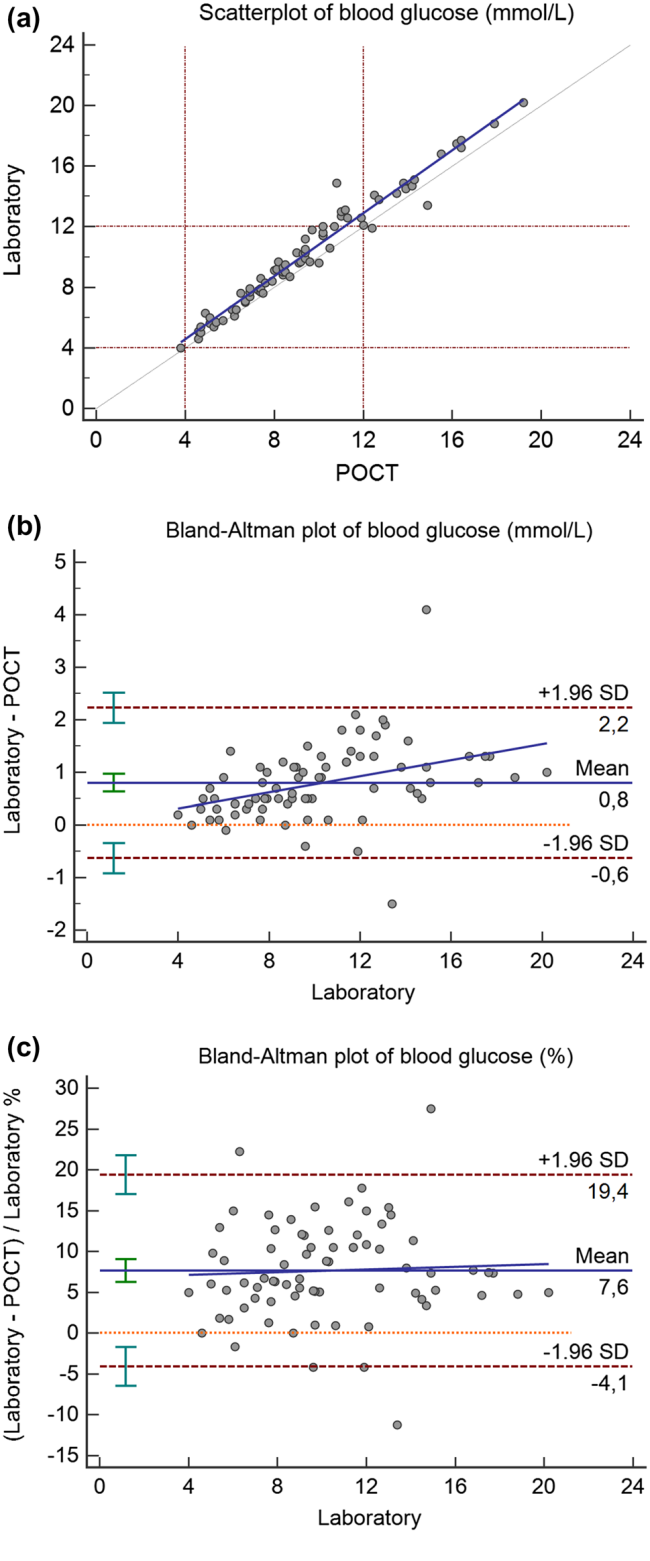
Comparison of POCT Accu Chek Inform II (ACI II) glucose meter with laboratory hexokinase method using arterial blood specimens. **a** Shows the comparison of the POCT ACI II with the laboratory hexokinase method measurements in arterial blood specimens during surgery in patients with DM under general anesthesia. **b** Presents the Bland–Altman plot of the same results in mmol/L. **c** Shows the Bland–Altman plot of these results in percentage

Graphical comparison of both measurements is presented in Fig. 1. Figure 1a shows results close to the 45° line of identity, with a tendency towards higher values with the laboratory analysis. The CCC was 0.955 (95% CI:0.933–0.970). Figure 1b shows the mean and random differences by means of a Bland–Altman plot. Mean difference (estimated bias) was 0.8 mmol/L and limits of agreement were -0.6 and 2.2 mmol/L. The ICC was 0.955 (95% CI 0.634–0.986), P < 0.001.

#### Hypoglycemic and hyperglycemic ranges

POCT ACI II and laboratory hexokinase method measurements both reported hypoglycemia (≤ 4.0 mmol/L) in one blood sample (1%). Hyperglycemia (≥ 12 mmol/L) was found in 16 (21%) blood samples with POCT measurements and in 23 (31%) blood samples with the laboratory method.

A total of 8 blood samples (11%) showed a difference of ≥ 15% (15.0—27,5%) between the two methods of measurement. These patients had a lower POCT than laboratory value.

#### Categorization

As shown in the alignment and traceability matrix (Table 3), which represents the use in clinical practice, 14 (19%) POCT ACI II measurements would have resulted in a different insulin dose adjustment, all by only one category. Each category gives a small adjustment (increase or decrease) in insulin therapy (NovoRAPID), depending on glucose concentration measurement. Because most POCT BG measurements were lower than the laboratory hexokinase method measurements, the change in insulin therapy resulted most often in a lower insulin infusion rate. Weighted Cohen’s Kappa was 0.82 (95%CI 0.74–0.91).

**Table 3:**
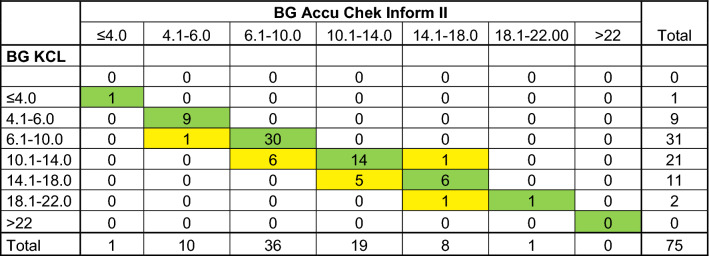
Alignment and traceability matrix [5]

## Discussion

In this study we investigated the reliability of Accu-Chek Inform II POCT blood glucose meter (ACI II) compared to a laboratory hexokinase reference method for glucose measurements on arterial blood in patients during non-cardiac surgery under general anesthesia, to examine the safe use of a POCT BG meter in these conditions. This study showed a CCC of 0.95 and ICC of 0.96 between the ACI II and clinical laboratory measurements. These values can be interpreted as substantial and excellent reliability.

Almost one fifth (19%) of the POCT glucose measurements indicated insulin dose adjustments which differed from corresponding adjustments according to laboratory values. However, these differences were small, and would not have resulted in major glucose shifts during surgery. Nevertheless, it remains important to regularly monitor BG to improve a patient’s glucose concentration.

Eight measurements (11%) showed a difference ≥ 15%, which is for glucose treatment an unacceptable deviation [10]. They all had lower POCT BG measurements than the laboratory hexokinase method measurements. There was no correlation between demographics, body temperature (absolute or fluctuations), medication, type of diabetes, and difference in collection of blood sample.

Based on our results, we recommend that whenever a POCT measurement is obtained that lies below the critical low threshold (4.0 mmol/L) or above the critical high threshold (12.0 mmol/L), an immediate laboratory measurement is done to check the POCT measurement.

In general, the performance of POCT-BG meters during general anesthesia could be influenced by the hematocrit, the blood pH, the temperature, and drugs.

During surgery, the hematocrit may fluctuate. This leads to a difference in viscosity and mechanical impedance of plasma diffusion, and thus glucose diffusion, into the reagent layer of the test strip. In case of some POCT meters this could give an overestimate of glucose in case of anemia and an underestimate in case of polycythemia [11]. Glucose measurements are based on one of three enzymes: glucose oxidase (GO), glucose-1-dehydrogenase (GDH), or hexokinase. For our study we have chosen to use the Roche, ACI II, which is a GDH-based, electrochemical measurement. It is therefore not susceptible to the extremes of hydration and oxygenation as a GO-based measurement would be. These extremes frequently occur during general anesthesia. The ACI II is a POCT which is not affected by hematocrit fluctuations, as it utilizes a correction for the hematocrit. Several solutions have been produced to correct for hematocrit influences (e.g. filter whole blood, or measure hematocrit). The Roche ACI II is not influenced by the hematocrit (broad range) when measuring the plasma-like value. If the viscosity caused by a high hematocrit in whole blood is too high, the Roche ACI II will produce an alarm. Other POCT glucose meters might be influenced by a high hematocrit without raising an alarm, however [12].

Since POCT glucose measurements are based on enzymatic reactions the results may also be affected by changes in pH. Limited studies on pH changes have been performed by others. These studies did not show major errors within a pH range of 6.95–7.84 [13, 14]. Since almost all patients who undergo surgery will have a pH in this range, we consider the pH level not relevant for this study.

Some studies suggest that low skin temperatures (15.5 °C) and low environmental temperatures (8 °C) may produce unreliable results [15, 16]. The effects of fever are unknown [2]. General anesthesia is a known risk factor for hypothermia, when no preventive measures are taken. The environmental temperature in our operating rooms was never below 18 °C and our active warming protocol keeps the patient’s temperature above 35 °C. In general, active warming may improve the accuracy of the measurements and can be an important factor for obtaining reliable results from the POCT BG.

General anesthesia cannot take place without the administration of drugs. Multiple drugs, such as acetaminophen, dopamine and mannitol are known to increase or decrease POCT BG readings, depending on the type of device that is used [17–20]. In our hospital, acetaminophen is used as premedication. However, acetaminophen may also affect the results of whole blood bench analyzers and is especially a problem in acetaminophen overdose [17, 19]. Since the patients received no acetaminophen during the surgery, no intoxication took place in this study population. Other drugs used in this study (for induction of anesthesia, pain-medication, anti-emetics, etc.) have no effect on the POCT BG measurements or are unknown to have an effect [19].

Another point is that, in general, laboratory measurement could be influenced by glycolysis. In this study POCT BG measurement and blood collection in the tube was done immediately after sampling. Also, the latest technology of sampling tubes was used to prevent glycolysis. Therefore, we consider it unlikely that glycolysis affected the laboratory measurements [8]. For this study we chose arterial blood samples, as lower glucose values can be expected in capillary/venous blood due to less interstitial refreshment because of limited blood flow. Limited capillary blood flow may cause deviant glucose values if capillary blood is obtained from extremities, such as the finger, because that blood is less refreshed or because of the pressure applied to the distal finger part to obtain the blood for a measurement. However, not all patients require an arterial catheter intraoperatively. Therefore, future research of the POCT BG meter with venous and/or capillary blood is required to assure safe usage intraoperatively. Also, other glucose measurement methods, e.g. flash glucose monitoring or real time continuous glucose monitoring, will require additional research when applied intraoperatively.

## Conclusions

Based on the present study, we conclude that BG measurements of arterial blood samples during general anesthesia with POCT BG ACI II meter are of no clinically relevant difference from standard clinical laboratory analysis. However, we recommend that whenever a POCT measurement lies below (4.0 mmol/L) or above (12.0 mmol/L) the critical threshold an immediate laboratory measurement is done to check the POCT measurement.

The results obtained in this study in arterial blood samples might not be generalizable to capillary/venous blood samples.

## Abbreviations

ACI II: Accu-Chek Inform II
BG: Blood glucose
DM: Diabetes mellitus
POCT: Point-of-care test
ICC: Intra-class correlation coefficient
CCC: Concordance correlation coefficient
GO: Glucose oxidase
GDH: Glucose-1-dehydrogenase
NIST: National institute of standards and technology
